# Urgent Surgery

**Published:** 1915-06

**Authors:** 


**Urgent Surgery.** By Felix Lejars. Translated from the Seventh French Edition by W. S. Dickie. Pp. xiv., 614. Bristol: John Wright & Co. 1914.

We can repeat our former commendation of the earlier edition. It has the stamp of marked individuality, is very practical, and the illustrations are highly artistic. The addition of chapters on acute dilatation of the stomach, acute pancreatitis, mesenteric obstruction, and sigmoiditis is a welcome one, and we are not surprised that so many editions have been called for. The valvular compression of the cardiac end of the stomach in acute dilatation is well illustrated, as well as the effect of posture in its relief. The routine division of muscle in operations for appendicitis is open to serious criticism, and we would like to see the grid-iron incision illustrated and described in future editions of a book by one who is master of his work.

